# To Remove Powder Stains

**Published:** 1890-04

**Authors:** 


### TO REMOVE POWDER STAINS.

Such blemishes are oftentimes so unsightly or disfiguring that any remedy that will remove them is worth remembering. According to *Medical Chips*, Dr. Ohmann-Dumesuil says that by painting powder stains with the following solution they will turn red:

℞. Ammonii biniodidi

Aquæ destillatæ - - - aa ℥j. Mix.

Then, if these red stains or marks are painted with dilute hydrochloric acid, they will disappear.—*Virginia Medical Monthly.*

